# A technology-based intervention to improve safety, mental health and empowerment outcomes for immigrant women with intimate partner violence experiences: it’s weWomen plus sequential multiple assignment randomized trial (SMART) protocol

**DOI:** 10.1186/s12889-021-11930-2

**Published:** 2021-10-28

**Authors:** Bushra Sabri, Nancy Glass, Sarah Murray, Nancy Perrin, James R. Case, Jacquelyn C. Campbell

**Affiliations:** 1 School of Nursing, Johns Hopkins University, 525 North Wolfe Street, Room S408, Baltimore, MD 21205 USA; 2 Nancy Glass, Johns Hopkins University School of Nursing, Baltimore, MD 21205 USA; 3 Sarah Murray, Johns Hopkins Bloomberg School of Public Health, Johns Hopkins University, Baltimore, MD 21205 USA; 4 Nancy Perrin, Johns Hopkins University School of Nursing, Baltimore, MD 21205 USA; 5 James R. Case, Johns Hopkins University School of Nursing, Baltimore, MD 21205 USA; 6 Jacquelyn C. Campbell, Johns Hopkins University School of Nursing, Baltimore, MD 21205 USA

**Keywords:** Intimate partner violence, Immigrant, Safety planning, SMART, PTSD, Depression, Empowerment

## Abstract

**Background:**

Intimate partner violence (IPV) disproportionately affects immigrant women, an understudied and underserved population in need for evidence-based rigorously evaluated culturally competent interventions that can effectively address their health and safety needs.

**Methods:**

This study uses a sequential, multiple assignment, randomized trial (SMART) design to rigorously evaluate an adaptive, trauma-informed, culturally tailored technology-delivered intervention tailored to the needs of immigrant women who have experienced IPV. In the first stage randomization, participants are randomly assigned to an online safety decision and planning or a usual care control arm and safety, mental health and empowerment outcomes are assessed at 3-, 6- and 12-months post-baseline. For the second stage randomization, women who do not report significant improvements in safety (i.e., reduction in IPV) and empowerment from baseline to 3 months follow up (i.e., non-responders) are re- randomized to safety and empowerment strategies delivered via text only or a combination of text and phone calls with trained advocates. Data on outcomes (safety, mental health, and empowerment) for early non-responders is assessed at 6 and 12 months post re-randomization.

**Discussion:**

The study’s SMART design provides an opportunity to implement and evaluate an individualized intervention protocol for immigrant women based on their response to type or intensity of intervention. The findings will be useful for identifying what works for whom and characteristics of participants needing a particular type or intensity level of intervention for improved outcomes. If found to be effective, the study will result in an evidence-based trauma-informed culturally tailored technology-based safety decision and planning intervention for immigrant survivors of IPV that can be implemented by practitioners serving immigrant women in diverse settings.

**Trial registration:**

This trial was registered with ClinicalTrials.gov as NCT04098276 on September 13, 2019.

## Background

Intimate partner violence (IPV) is a serious public health problem disproportionately affecting immigrant women in the United States (U.S.). The prevalence of IPV in community-based samples of immigrant women in the US range from 17 to 70.5% [1]. Foreign-born immigrant women in abusive relationships are at high risk of severe violence or homicide perpetrated by a male partner [2]. Factors such as minority racial/ethnic identity, immigration status and low socioeconomic background may limit immigrant women’s access to resources, constrain help seeking behaviors, and diminish their overall safety, health, and well-being [3]. Further, immigrant survivors of IPV may not seek help due to lack of knowledge of the U.S. system, undocumented status, stigma, and shame associated with experiencing IPV and a lack of social support [4]. Inability or unwillingness to seek resources or support may place immigrant women at greater risk of repeat IPV, severe IPV, and partner homicide. Thus, there is a need for timely and effective safety planning interventions that identify at-risk immigrant women in abusive relationships and connect them with services to enhance safety. Further, safety planning could help survivors enact behaviors to enhance their safety and minimize harm while in the relationship or when separated from the abusive partner [3, 5]. Safety planning is critical for women in abusive relationships as abuse can escalate over time, increasing the risk of serious harm such as injury or homicide.

Safety planning interventions use strategies that increase situational awareness of IPV-related risks among women and empower women with skills necessary to enhance safety [5]. The safety planning process begins with understanding a woman’s perspective of her situation, including her assessment of risks, then gathering relevant information, assessing past and current safety strategies, evaluating the current situation, and identifying the types of support and resources needed, before developing a plan to prevent and address IPV [6, 7]. Since there are unique risk and protective factors for IPV experienced by immigrant women (e.g., documentation status), their safety planning needs may differ from non-immigrant women [3]. For instance, in a qualitative study with immigrant women survivors of IPV, women highlighted a need for culturally informed safety planning that integrates their unique needs as immigrants (e.g., making services available without use of an ID, orienting women to laws and rights in the US) [3, 4]. In some immigrant communities, women face abuse from both partners and in-laws. For these women, there is need for additional safety planning around their families [3, 4].

Technology-based interventions, i.e., digital interventions that can be implemented via computer/web-based platform, mobile device, or online social media [8, 9], offer a feasible and acceptable alternative to improve remote access to safety planning for immigrant women who experience IPV. These interventions can address barriers women face by providing remote support through technology, building women’s knowledge and awareness of available resources, remotely working with women to develop safety plans, and enhancing access to care. By not necessitating face-to-face interactions in person, technology-based interventions can afford greater privacy and thus reduce exposure to stigma [10–13]. This is essential as known barriers to in-person service use include not only geographical distance, but also fear of experiencing shame, judgment, or general discomfort with discussing sensitive topics during in-person interactions [14–17].

Although some immigrant women may face barriers to accessing technology due to limited access, evidence indicates smartphones and other personnel devices with access to internet are important to social connectivity among immigrant populations. In 2015, up to 74% of Hispanic/Latino, including immigrants reported using the internet, with mobile phones as their primary means of internet access [18]. In a study by the Pew Hispanic Center in 2010, over three quarters of the foreign-born Latino immigrants used cell phones (89%) to communicate with family and friends. Many immigrant women use smartphones for communicating with friends and families in their host countries. The internet is the most cost-effective way of calling long-distance by phone. Various apps (e.g., Whatsapp) make it easier for people to communicate long distance with no extra cost for calls or text messages. Smartphones and apps are also used to transfer funds to families in home countries. Several studies similarly note the importance of mobile phones/internet for immigrant populations, even if device access is sporadic for financial purposes [19, 20].

Despite the potential of technology-based support for survivors of IPV, immigrant survivors of IPV remain an understudied and underserved population in need of rigorously evaluated, evidence-based and culturally informed technology-based interventions that address their health and safety needs. Further, a “one-size fits all” approach to interventions or a single intervention may be limited in effectiveness due to heterogeneity in intervention response. Therefore, our study uses an adaptive sequential, multiple assignment, and randomized trial (SMART) design to evaluate a technology-based intervention called “weWomen Plus” tailored to the needs of immigrant women with IPV experiences. SMART design is a methodologically rigorous way to optimize interventions and maximize their utility and implementation in real-world settings [21, 22]. An adaptive intervention is defined as an intervention which adapts the type or dosage of an intervention based on participants’ characteristics or responses [23]. An adaptive SMART design allows for more precision in identifying dose and types of intervention components that can result in best outcomes. This often involves increasing the intervention intensity or augmentation with another intervention in some way if participants fail to meet specified benchmarks for improvement or response. Immigrant women may differ in their responses (i.e., outcomes of the intervention) to one type of technology or intervention due to their varying intervention needs. Some women may respond whereas others may not respond to the same intervention type or intensity due to factors such as fear of husband or his extended family or lack of social support. An adaptive intervention approach, is therefore, needed to provide needs-based intervention to abused immigrant women.

### Description of the weWomen plus intervention

The weWomen Plus intervention is an adaptive technology based (online/web-based, text and phone) trauma-informed culturally tailored intervention designed to reduce the risk of future IPV or a homicide, improve mental health and increase empowerment of immigrant women in abusive relationships. The online/web-based component of weWomen Plus is the culturally adapted myPlan intervention (described in the next section). This study will evaluate the relative effectiveness of the culturally adapted myPlan app alone, and for initial non-responders, in combination with additional safety and empowerment strategies delivered via text messages and/or phone calls on outcomes of safety, health and empowerment, as compared to usual safety planning over 12 months with immigrant women. Although phone and text intervention strategies have been used in other intervention studies (e.g., HIV risk reduction) and with other populations (e.g., African American young adults), no studies examined use of text and phone calls as part of an IPV intervention for immigrant survivors of IPV. Further, these strategies have not been culturally tailored and tested for immigrant survivors of IPV in a SMART trial in prior research. Given the high need and specific barriers to accessing existing interventions experienced by immigrant women, this study seeks to provide evidence-based technology-delivered strategies that can be employed in multiple service settings to improve the health and safety of diverse immigrant women who experience IPV in the US.

#### myPlan app, an evidence and technology-based safety decision and planning intervention

MyPlan is a safety decision and planning tool delivered through a free and secure web-based app. The myPlan intervention was designed based on Dutton’s empowerment model [24] to facilitate and support: (a) protection, such as understanding of severity/danger in the relationship using the validated Danger Assessment (DA); b) decision-making, by identifying safety priorities (e.g., protecting children) thus reducing decisional conflict; and, (c) healing from the negative health and social effects of IPV, e.g., by encouraging use of tailored safety strategies and providing links to resources and community-based services. Prior to downloading and using the myPlan app, an on-boarding section provides the user with information on safe technology use (e.g., not sharing passwords, not using myPlan on a device to which an abusive partner has access, etc.), privacy and requests that they create a secure 4-digit code as a password to enter the app. Once the on-boarding is complete, all users immediately receive a safety plan that provides strategies such as calling an advocate at a national or state hotline, packing a bag in case they need to escape quickly, and hiding away money and important papers; however, these safety strategies are not tailored to the user’s specific situation. Users are therefore then invited to complete an additional three sections within the app to tailor the safety plan to their situation, priority, and needs. In the first of these sections, users are provided information on how to identify “red flags” (e.g., jealousy, controlling behavior, isolation from friends/family) of an unhealthy relationship. Next, the users complete the Danger Assessment (DA). The DA, previously adapted and validated for immigrant women in the US, consists of 20 yes/no questions on factors known to increase risk of severe and lethal violence in an abusive relationship (e.g., increase in frequency and severity of IPV over past year, abuse during pregnancy, threats with a weapon). Based on their responses to the DA, women are categorized at the following levels of danger: variable (< 8), increased (8–13), severe (14–17) and extreme (> 18) danger. Immediate visual feedback on the danger score and its interpretation is provided to the users. Users are then taken through the third section which consists of a priority-setting activity where they can consider the information provided within the context of their needs and values (e.g., having resources, safety, and well-being of children) with regard to their relationships. Inputs from the users across the three sections are combined using an evidence-based algorithm to create a tailored safety plan that includes links to local (as available) and national resources and services.

The myPlan app intervention has been widely tested and found to be efficacious in reducing decisional conflict and increasing safety behaviors in survivors of IPV living in the US, Canada, Australia, New Zealand, and Kenya [12, 25–28]. In the most recent randomized controlled trial of myPlan among college students aged 18–24 years, women who received myPlan reported increased use of helpful safety strategies, reduced reproductive coercion and suicide risk relative to their peers that received usual safety planning resources [29]. While the myPlan intervention has demonstrated its effectiveness in improving safety related outcomes in multiple studies, our previous research using technology-based intervention [30] found that immigrant women may need different and additional intervention delivery mechanisms (e.g., text messages and phone calls with advocates) and higher dosages of the intervention to improve safety, health, and empowerment outcomes.

#### Text messaging intervention

As with the myPlan intervention, the text message intervention is based on a strengths-based, empowerment framework [24, 31] with sessions designed to empower women to take control of their safety while validating and respecting their choices and working with their priorities and needs. It also draws from a psychosocial readiness model [32], assessing for women’s readiness towards safety on a change continuum. Accounting for the level of readiness, the text sessions are designed to bolster immigrant women’s awareness of safety planning and available community services, to conduct check-ins on safety, to assess level of risk for severe or lethal violence in the relationship, identify ongoing barriers for implementing safety strategies, and to enhance self-efficacy for safety behaviors.

The tailored weekly messages are sent once a week for 4 weeks and at preferred and safe days and times designed by the woman. Table 1 details the content by each text session. Overall text sessions use empathetic tone in written communication, include words to enhance feeling of self-worth, and continually emphasize the importance of self-care. Every text session ends with a list of emergency resources and reminders to delete text messages from phone, etc. for safety.

**Table 1:** Description of text intervention

Week	Assessed	Information provided	Tailoring
1	• Women’s current feelings of safety • Level of readiness to take safety actions • Past use of safety strategies and barriers encountered • Barriers to implementing safety behaviors	• Elements of safety plans • Information on additional steps and resources for safety	Based on level of readiness
2	• Women’s current feelings of safety • Formal and informal support resources available for protection • Helpfulness of suggestions or resources provided in Week 1 • Need for additional resources	• Importance of self-care • Self-care tips	Based on whether the resources provided in the prior sessions were useful or if there is need for additional support in terms of safety
3	• Women’s level of danger (self-perception of future severe violence risk) from 1 to 10 ◦ If score > 6, receive short form of the DA to further assess their level of danger from the abusive partner • Safety risk of greatest concern	• Safety plan messages • Self-care strategies for poor mental and physical health • Service referrals	Based on indication of need for additional support in Week 2 and self-reported risk of danger, living situation and relationship status with partner (e.g. ended relationship but still living with partner, ended relationship, and living away from partner).
4	• Safety goals of relationship regardless of relationship status (including safety even when relationship has ended) • Any additional information regarding current situation	• Resources and services in the community	

#### Phone call intervention

The trauma-informed empowerment-focused phone intervention component uses a strengths-based perspective [31] in assisting women in recognizing and utilizing the strengths and resources that they may not recognize within themselves. Other strategies include motivational enhancement [33, 34] and solution-focused techniques [35]. The facilitators who speak with women on the phone are trained to use supportive communication that expresses empathy, validates women’s emotions, and identifies and highlights their protective factors and strengths (e.g., by reframing deficits with a new perspective that emphasizes accomplishment and positive traits) to support them towards healing and recovery. Safety assessment is part of both phone sessions. The first phone call takes place 1 week after the end of the text intervention, and the second phone call takes place 1 week after the first phone call. In the first phone call, the facilitator establishes rapport of trust, discusses primary concerns, emphasizes accomplishments, reframes negative thoughts to strengths, uses scaling questions to build motivation, elicits change talks, identifies modifiable changes that are within women’s control, and develops mutually identified, attainable goals that reflect women’s priorities. In the second phone call, the focus is on discussing steps taken towards goals, barriers to goals, discussing exceptions, identifying general and culturally specific strengths as well as assessing personal and community resources, and reinforcing goals and strategies discussed during sessions.

## Methods

### Research design and aims

**Fig. 1:**
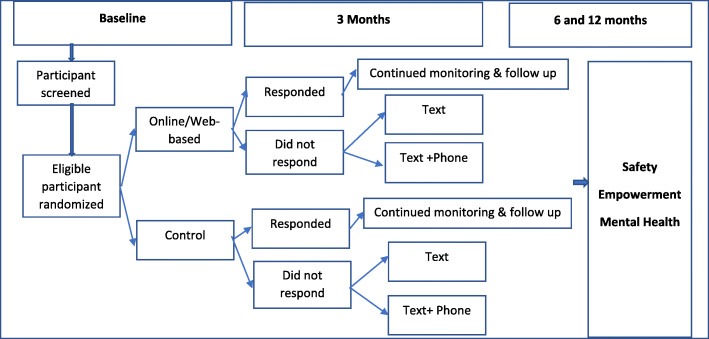
It’s weWomen plus SMART trial

This study uses an adaptive sequential, multiple assignment, and randomized trial (SMART) [21] design to evaluate the effect of weWomen Plus intervention on safety, mental health, and empowerment outcomes for immigrant survivors of IPV (Fig. 1). Our aims are a) to identify predictors of response to the online or web-based component of the intervention (the culturally adapted immigrant version of myPlan), focusing on outcomes of safety, mental health, and empowerment; b) to assess the relative effectiveness of supportive text messaging or a combination of text and phone support on safety, mental health, and empowerment outcomes among the non-responders to the online/web-based intervention i.e., (adapted immigrant version of the myPlan) alone. Women are initially randomized to receive the online/web-based (adapted myPlan intervention) or usual care (control) group. At 3 months, non-responders, defined as women who report both no improvement in safety (i.e., significant reduction in severity and frequency of IPV) and empowerment scores (i.e., significant improvement in empowerment) from baseline to 3 months, are re-randomized. Non-responders in the intervention and control arms are re-randomized to text messaging alone or a combination or text messaging and phone intervention. Responders continue in their originally assigned arms. We hypothesize that adding text messaging and phone call interventions will improve outcomes among the non-responders and immigrant women who receive a combination of text and phone intervention will show greater improvement in outcomes than those who receive text messaging alone. Further we hypothesize that the non-responder groups of women will increase to the level of the responders on safety and empowerment outcomes after participating in text and/or a combination of text and phone intervention.

### Study participants

In order to participate in the study, potential participants must: be 18 to 64 years of age; identify as a woman (assigned female at birth or have a gender identity as a woman); have been in an intimate relationship in the past 12 months; report having experienced IPV (physical, sexual or psychological) in the past 12 months; be a foreign-born immigrant living in the US; have access to a safe computer or a smartphone and be comfortable using it to access the study internet site; and be willing and able to be contacted by phone or text. Participants with no experience of IPV within the past year, are US-born, are younger than 18 or older than 64, those who cannot access or use internet or phone and are not willing to be contacted by phone or text are excluded.

### Study setting, recruitment and data collection procedures

Women are being actively recruited from multiple states across the US (e.g., Texas, New York, New Jersey, California, Minnesota, Massachusetts, Maryland, Virginia, and Washington DC) with no limitation by state for inclusion in the study. The study information is being disseminated through organizations that include immigrant-specific general service providers, and health clinics as well as immigrant IPV support agencies throughout the country. To reach the broadest range of participants, women are also being recruited through listservs and social media platforms (e.g., Facebook, Twitter, Instagram, etc.). Women are directed to the study website which provides information about study-related expectations, incentives, and eligibility. There, interested women can click on a link to complete an eligibility screener online. If found eligible, women are asked to provide their contact information- including a secure phone number and email address to which all study-related information is sent. The study team reviews eligible women’s registration information and communicate with them over the phone as a validation step (i.e., to avoid false or repeat registrations) before the participant is enrolled in the study and completes the baseline survey. The team members obtain verbal consent when speaking with participants. Participants also complete an electronic written consent when logging into the study website and are required to indicate consent prior to proceeding to the survey. Data is being collected through a web-based or smart phone application-based questionnaire using a Clinical Trials Management System (CTMS) depending upon the preference of the participant. Data collection occurs at baseline and at 3, 6 and 12 months follow up. All study team members receive standardized training before data collection. The training covers study implementation procedures, data management, and safety and ethical considerations when interacting with participants. This includes safe use of mobile devices and working with participants at risk for harm to themselves or others or with participants in immediate danger.

### Randomization and blinding

For first stage randomization, women who consent, enroll in the study, and complete the baseline survey are randomized using computer-generated block randomization stratified by length of time in the US---to either the online/web-based intervention website or the control website. Study team members are masked for the first stage randomization status. Randomization for the second sequence of the intervention is also computer generated but study team members are not masked to second stage randomization status of participants. For second stage randomization, women in the intervention and control arms who do not show reduced violence or improved empowerment (referred to as non-responders) at 3 months follow up survey are re-randomized to receive the text intervention or a combination of text and phone intervention. Based on women’s responses to the measures at the 3 months follow up, the scores for identifying responders and non-responders are automatically generated in the CTMS and the research team members are alerted for non-responder participants, the re-randomization arm women they are assigned to and the need to initiate the text and a combination of text and phone intervention. Women who do not show increased severity and frequency of IPV or show improved empowerment scores in both arms continue in the originally assigned arm and complete the follow up study measures.

### Intervention Fidelity

The training manual of study procedures covers the intervention protocol as well as recruitment and retention protocols. Research team members are trained in all participant contacts. We have a checklist for facilitator to ensure that the text and phone sessions are delivered as desired. Overall feedback and regular check-ins with participants also identify any problem in implementation of the intervention. Any technical difficulties with study websites or the text component are resolved immediately with support from our study programmer. Further, we address ongoing challenges in implementation through weekly team meetings.

### Description of the control condition

The control condition receives usual safety planning resources modeled on national and state domestic violence online resources. Women in the control condition are not administered the DA or provided with DA-informed tailored safety planning. However, women do complete risk assessment questions based on our formative work which identified additional risk factors for IPV not currently included in the DA [4] and receive standard safety planning information. Safety check-ins are part of all contacts with participants.

### Outcomes

The primary outcomes are:

- *Severity and frequency of IPV*: The adapted version of the Revised Conflict Tactics Scale (CTS2) [36] is used to measure severity and frequency of the abusive or violent acts in intimate partner relationships. The CTS2 subscales include physical aggression, injury, psychological aggression, and sexual coercion. Response categories range from 0 = never to 6 = more than 20 times within the past 12 months; 7 = not in referent period but happened before. Higher values on the measure within the past 12 months indicate severe or more frequent experiences of violence. The CTS-2 items are scored using the severity-times-frequency weighted score, as recommended by Straus [36].
- *Empowerment:* This includes assessment of overall empowerment and empowerment related to safety.
  i) *Overall empowerment*: The Personal Progress Scale-Revised (PPS-R; alpha = 0.88) [37] is a 28 item self-report measure of empowerment designed to assess multiple areas associated with empowerment such as positive self-evaluation, self-esteem, ability to regulate emotional distress, gender-role and cultural identity awareness, self-efficacy, self-care, problem-solving, assertiveness skills, and access to resources. Participants’ responses are rated on a 7-point scale ranging from 1 (Almost Never) to 7 (Almost Always). The items are summed to create a total score for empowerment. The range of scores is 28–196 with higher scores indicating a greater degree of empowerment.
  ii) *Empowerment related to safety:* The empowerment related to safety is assessed by the Measure of Victim Empowerment Related to Safety (MOVERS) scale. The MOVERS (alpha = 0.81) [38] is a 13-item scale that measures empowerment within the domain of safety (e.g., extent to which a participant has developed a set of safety-related goals and a belief in her ability to accomplish them, the extent to which she feels that her efforts to achieve safety trigger new difficulties and extent to which she has knowledge about and access to support). Participants respond to each item using a five-point scale from “1 = never true” to “5 = always true”. The scores on the measure are summed and averaged to produce a total score. The scores range from 13 to 65 with higher scores indicating a greater degree of empowerment related to safety.

The secondary outcomes are

- *Depression:* The Patient Health Questionnaire (PHQ-9; alpha = 0.89) [39] is a 9-item measure to assess past 2 weeks depression symptoms based on the diagnostic criteria for major depressive disorder in the Diagnostic and Statistical Manual (DSM-IV). Each of the 9 items score from 0 (not at all) to 3 (nearly every day). A total score is computed to measure severity of depression by summing the items. Higher scores indicate more severe depression symptoms.
- *Post-traumatic stress disorder (PTSD):* The Harvard Trauma Questionnaire (16 items; alpha = 0.96) [40, 41] is used to measure past week symptoms of PTSD derived from the DSM-IIR/DSM-IV criteria for PTSD, with response options ranging from 1 (Not at all) to 4 (Extremely). The items represent intrusion/re-experiencing, avoidance/numbing and hypervigilance/arousal symptom clusters. Higher scores indicate more severe PTSD symptoms.

### Retention measures

The study has a robust retention plan to maximize participant retention over the 12 months period. Women are compensated for their time completing the surveys ($40 per survey with the possibility of a total of $160 in compensation over the course of participation in the study). At enrollment, women are asked to provide phone numbers and emails of at least two alternate safe contacts in case we are unable to contact them using the number and/or email they provided. An automated email is sent to women’s email addresses 2 weeks before, 1 week before, and on the day each follow-up survey is scheduled. The study team members directly reach out to women who do not complete a follow up assessment on schedule by their preferred mode of communication and day and time indicated at baseline. Women are also contacted every month when there is not a follow up assessment scheduled through the course of the study. These monthly contacts are designed to conduct brief check ins, confirm their contact information, and discuss any changes to their preferred mode of contact. These retention check-ins do not occur with non-responders while they are receiving the text and/or phone interventions.

### Data analyses

T-test and chi-square tests will be used to examine if each randomization succeeded in creating balance on key demographic and outcome variables assessed at baseline. We will control for any variables that are found to be significantly different between the intervention and control arms in the main analyses. The distribution of all outcome variables will be examined to determine if they meet the assumptions of planned analyses. Those with and without missing data will be compared on baseline variables to determine variables related to missingness. Variables related to missingness will be included in the analyses which should yield valid inferences [42]. Missing data will be handled with multiple imputation techniques with the Bayesian based Markov-chain Monte-Carlo method. A total of 15 imputations will be specified and pooled estimates will then be reported for all subsequent analyses. The main analyses will be conducted under intention-to-treat principles.

To assess the relative effectiveness of weWomen Plus intervention versus control on safety, empowerment and mental health outcomes, Generalized Estimating Equations (GEE) with robust variance estimation will be used. The parameter of interest will be the group by time interaction which will determine if the change over time in the outcome variables differs between the two groups. We will also identify baseline variables related to response/non-response to the online/web-based component among those randomly assigned to the online/web-based intervention group in the first stage randomization using logistic regression. We will examine 2 indicators of response (safety and empowerment). For safety and empowerment, non-response will be defined as less than a 0.25 standard deviation improvement in scores (decrease in IPV and increase in empowerment) from baseline to 3 months. Predictors of response will include demographics (e.g., age, race, marital status, and time in the US) and other relevant variables such as acculturation, attitudes towards violence against women, and social support. Both bivariate and multivariate models will be used to examine each individual variable’s relationships with non-response and to examine the relative importance of the predictors of non-response. These analyses will be used to identify covariates for the main intervention evaluation analyses after second stage randomization.

GEE will also be used to determine if adding text messaging and combination of text messaging and phone components brought the non-responders to the level of the responders on key outcomes. The analysis will compare three groups: 1) responders to the usual care control arm and responders to online/web-based arm, 2) woman re-randomized to the text messaging, and 3) women re-randomized to text messaging + phone contact. The GEE model will include time (baseline and 12 months), group, and the group by time interaction as the parameter of interest to determine if the change over time in the outcomes differs between the responders and those receiving adaptive intervention components. We will use the ‘weighted and replicated’ estimation method [43] so that effects for the adaptive interventions can be simultaneously estimated in the same model. Inverse probability weighting for probability of receiving an adaptive intervention will be relative to the number of times an individual is randomized (e.g., responders have a weight of ½ and non-responders have a weight of ¼). As observations need to be used in more than one comparison, data will be replicated. Sensitivity analyses will include baseline variables found to predict intervention response at 3 months as covariates in the models.

### Statistical power

Based on our prior work, we estimate 30% of women to be non-responders to the online/web-based component*.* Assuming an 80% retention rate at 12 months, we determined the sample per group for study aims needed to detect an effect is of .25 or greater. Compared to text, we hypothesize that the phone-based intervention will allow for more rapport building and personalized care through interaction with a trained interventionist using strengths-based motivational interviewing and solution focused approaches, so we expect an effect size in the small to moderate range (Cohen’s d of .2–.4) when comparing the combination of text and phone to text only intervention arms. Interventions drawing from SMART design/SMART trials may be powered similar to a standard RCT, if stage-specific questions (e.g., comparison of first-stage interventions averaging over response and second-stage interventions) are of primary interest and embedded dynamic treatment regimen development is of secondary interest [44]. With power = 0.80, alpha = 0.05, and *N* = 253 per group, we will be able to detect significant differences in the change over time between the two groups when the effect size is 0.25 or greater. This will require a total sample size of *N* = 1266 with 633 randomized to online/web-based and 633 to usual care. With a 30% nonresponse rate, 190 women will be eligible to be randomized to the online/web-based +text or online/web-based + text messaging + phone with 95 per group. We expect a higher rate of non-responders in the control arm, with 50% of women (*N* = 316) eligible for randomization to text messaging only or text +phone with *N* = 158 per group.

### Risks and safety

#### Protection against risk

All women complete an online informed consent process prior to enrollment, in which they are provided with information on the study purpose and potential risks in participating: loss of confidentiality, distress, fatigue, and potential retaliation from the partner if they learn of study participation. The safety protocols for the study are developed to minimize these risks*.* All study team members completed a mandatory training on protections against risk, as well as training on all study and safety protocols. This included sensitization to the experience of abused women and safety issues they may face, how to minimize risk to women’s safety during interactions with the study team, and issues related to informed consent.

Specific steps taken to protect participant safety and confidentiality include the use of study code numbers for identification, reporting of aggregate data, omitting identifiers in the data collected and maintaining contact information separately from data, and destroying all contact information within 3 years after completion of the study. No information about woman’s participation is given out to anyone outside the research team. To ensure safety in data collection, women are provided a username, password, and a pin to access the survey and intervention sessions. Women are provided with safety instructions such as using a computer which partner cannot access, accessing private browsing and deleting any text communication. Women are contacted using the preferred method and preferred times indicated at baseline to help prevent inadvertent disclosure of participation that could compromise her safety.

Implementing important safety procedures for internet, phone, and text communication as well as for handling study data serve to protect women from harm. During the study, women may stop at any time. The team conducts periodic safety check ins and provides information on safety resources as well as strategies to safely use internet and phone. No information about woman’s participation is given out to anyone outside the research team. To ensure safety in data collection, women are provided a username, password, and a pin to access the survey and intervention sessions. Women are provided with safety instructions such as using a computer which partner cannot access, accessing private browsing and deleting any text communication. The study has an established suicide protocol which includes an algorithm for assessment, maintaining contact with participant, accessing study investigators and consultants for support and follow-up, accessing community suicide hotlines, and connecting the participants to the trained counselors. Should a research team member discover child abuse, suicide or homicide intent, a report to the appropriate agency will be made, and this is included in study consent forms. *F*urther, the study team has a plan for documenting adverse events, action taken and follow-up procedures for the action. If any adverse event is identified by the study staff, it will be reported directly to the Principal Investigator, the Data Safety Monitoring Board (DSMB). Adverse events will also be reported to the Johns Hopkins University (JHU) Institutional Review Board (IRB) according to the policy of the JHU IRB using the standard Protocol Event Report form in the electronic IRB system. All adverse events will be tracked and incorporated in the evaluation of study safety procedures.

#### Safety procedures to ensure data privacy

For web-based measures, enrolled women are given a username and password to access a secure online survey application that allow them to self-report and store their de-identified data. This system uses usernames that do not identify women, but simply allow them to have a unique identifier to access the system. When the member of the research team is contacted by an interested woman, the team member logs into the tracking system and creates a new screening record for the interested woman after collecting identifying information. The system creates a unique subject ID for the woman along with a password, which is sent to the woman via email. The actual identifying information of the woman is recorded by the research team member in a different system to be used just for tracking the study participants. The institution’s hosted system is also username and password protected with SSL encryption enabled to ensure all transactions over HTTP are not decipherable. The system logs all access attempts and sessions for any authorized user and is designed to collect study participant identifying information as well as their schedule and progress with the study. On enrolling a woman into the study, the system generates a unique, non-identifying study ID and password which is used by the woman to access the self-reporting system. At the termination of the study and after analysis has been completed, a copy of the de-identified data set will persist as a file only. The transactional databases used to store personal information and study information will both be deleted. There will be no key to link any of the de-identified records to any identifiable individual. All persons with access to data (i.e., research team members) will rigorously follow procedures to ensure confidentiality of data. Only authorized study staff can log on or access the master study database.

#### Data safety and monitoring board

The study has a Data Safety Monitoring Board (DSMB) comprised of three members. The members are experts in ethics or responsible conduct of research, intervention research, biostatistics, and in work with immigrants and survivors of IPV. The members are independent of the study or investigators and have no competing interests related to the study or the intervention trial. The DSMB is responsible for ensuring that a) participants are not being exposed to unnecessary or unreasonable risks as a result of the pursuit of the study’s scientific objectives and b) all study and intervention protocols to ensure safety are adhered to consistently. At the 6-month intervals, interim analysis reports are provided to the DSMB along with any other analyses that the members may request for safety monitoring. The DSMB can make recommendations about continuing or stopping the trial based on safety or other concerns.

### Dissemination and data sharing plan

The dissemination plan is based upon the National Institute of Health (NIH) Policy on the Dissemination of NIH-funded clinical trial information (https://grants.nih.gov/policy/clinical-trials/reporting/understanding/nih-policy.htm). The study findings will be available to the community of scientists or researchers interested in abused immigrant women’s safety, health, and empowerment. The findings would also be available to policy makers, domestic violence and sexual assault advocates, legal advocates, healthcare, and other service providers. The collaborators on the study can request findings from the study that are aggregated to the state level. The results will be disseminated through presentations in local, national, and international conferences and through peer-reviewed publications. The authorship will be based on contribution to the study elements and the publication. A password protected de-identified database will be created to enable planned analyses for publications and presentations as well as for the final report. Only the study team members will have access to the database for analysis.

## Discussion

Immigrant women are a high-risk group for IPV. However, there are no rigorously evaluated interventions to improve safety, mental health, and empowerment outcomes for abused immigrant women, who most likely remain at high risk of revictimization and negative effects of IPV and are underserved. Further, a single intervention approach may not address the heterogeneous intervention needs of immigrant survivors of IPV who come from diverse backgrounds and cultures. Due to their varying needs, all immigrant women may not experience improvement in outcomes by an intervention at the same intensity level. Immigrant women unable to implement safety plan on their own using an online platform may benefit from additional support by phone focusing on other strategies such as strategies to increase support from other immigrant women and specific strategies aimed at their abusive partners. To the best of our knowledge, this is the first study that is implementing and evaluating an adaptive trauma-informed culturally tailored technology-based intervention (web-based, text and phone) for immigrant women in the US, using a SMART trial. Using rigorous methods, this study will address unmet needs of immigrant women by providing additional intervention support for those who do not show positive outcomes by one type of intervention. The findings will show the utility of technology-based approaches for working with immigrant survivors who often face barriers in receiving help through in-person interventions. If found to show positive outcomes, this technology-based intervention can be used in a variety of settings such as healthcare settings, social service organizations and domestic violence agencies serving immigrant women. The intervention can also be useful for women who are not seeking services and can help practitioners to provide more culturally informed services. For primary care clinics or other organizations that are not focused on IPV, this will be a useful tool for them to address needs of immigrant IPV survivors. Findings of this study will support the immigrant health initiative which focuses on designing and implementing effective interventions to reduce the health disparities among immigrant populations and address health inequity issues (e.g., IPV and lack of safety).

## Data Availability

Not applicable.

## Abbreviations

IPV: Intimate partner violence
PTSD: Post-traumatic stress disorder
DA: Danger assessment
PHQ-9: Patient Health Questionnaire (PHQ)-9
MOVERS: Measure of Victim Empowerment Related to Safety
CTS-2: Revised Conflicts and Tactics Scale
IRB: Institutional Review Board
SMART: Sequential, multiple assignment, and randomized trial
GEE: Generalized estimating equations
RCT: Randomized controlled trial
US: United States
